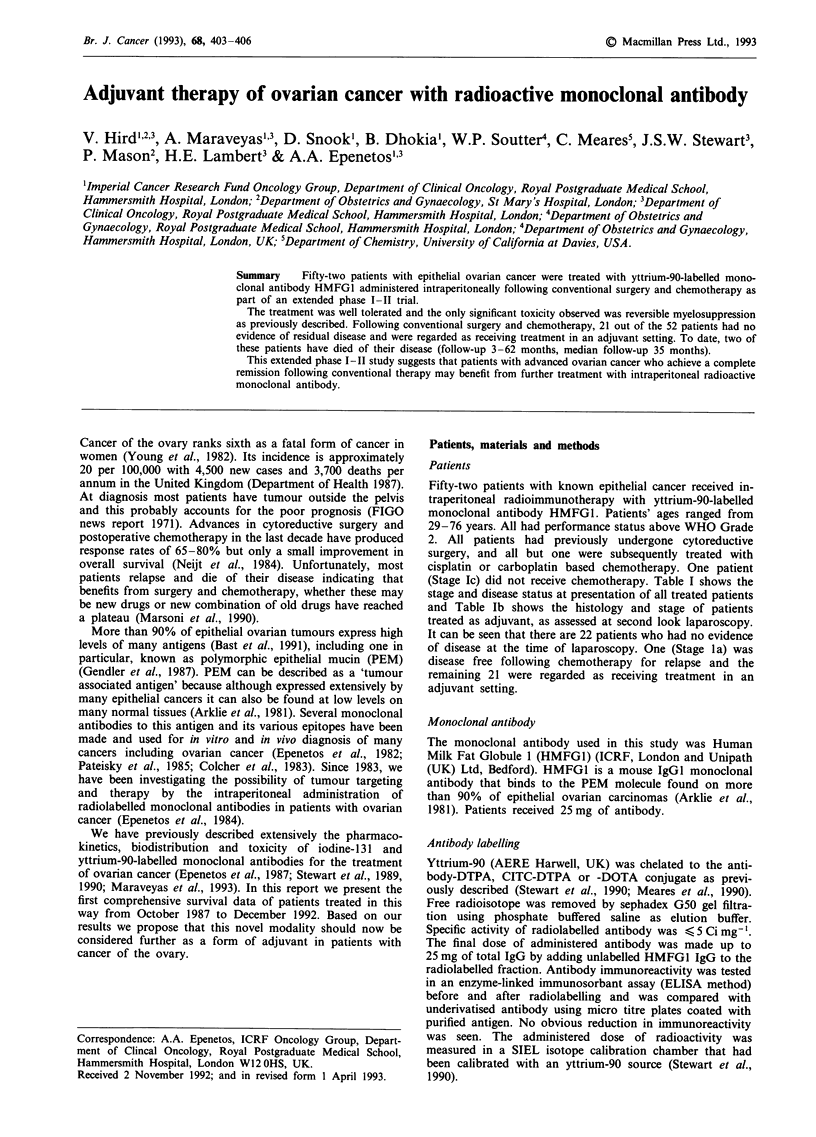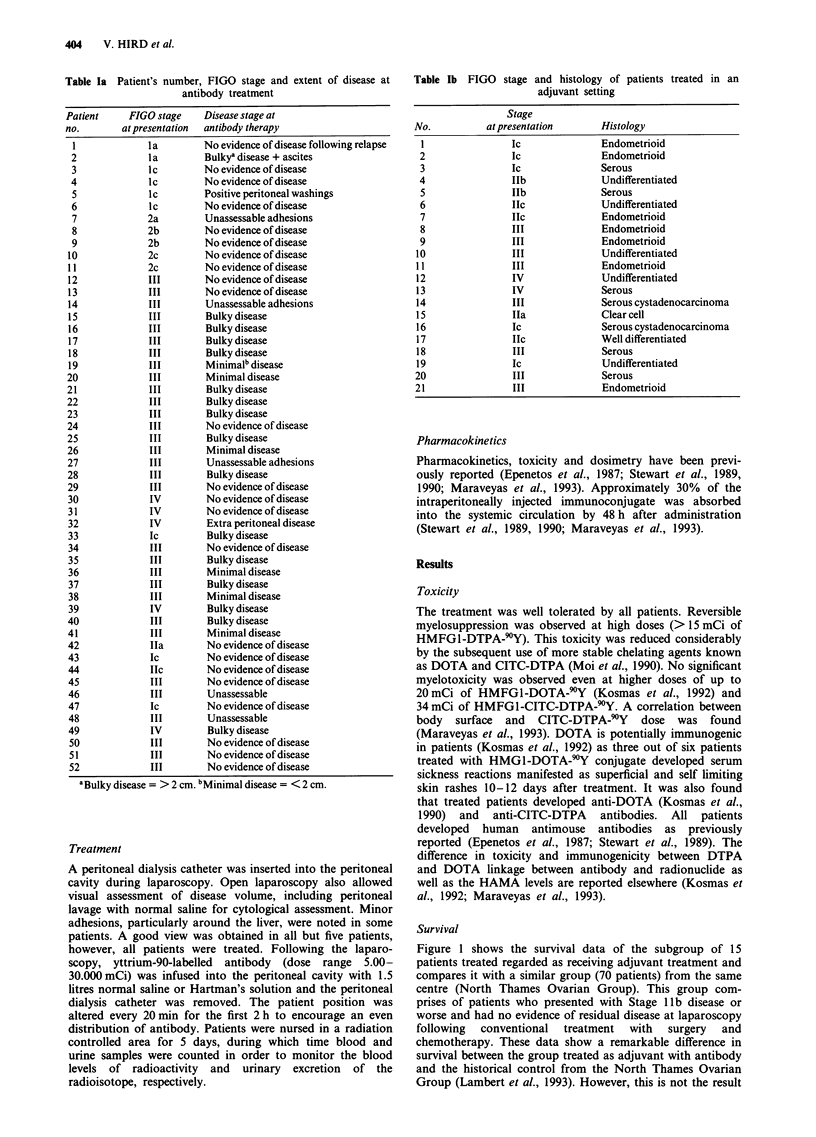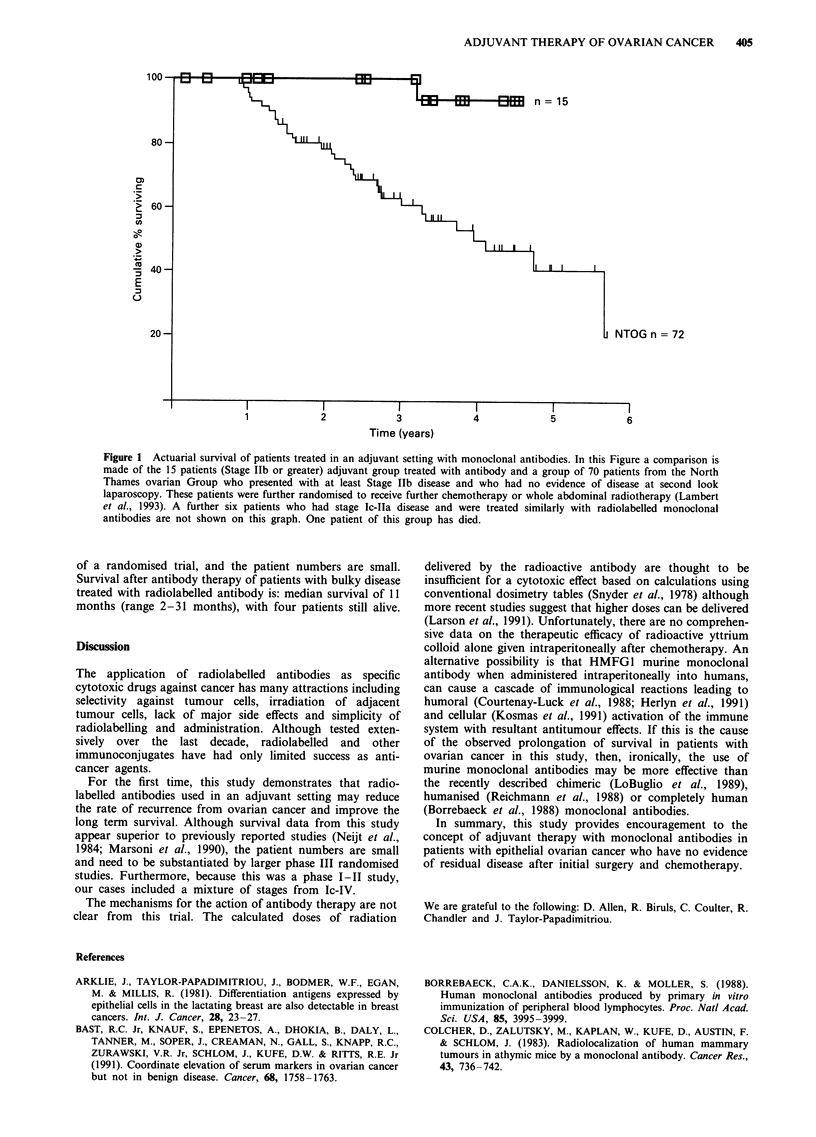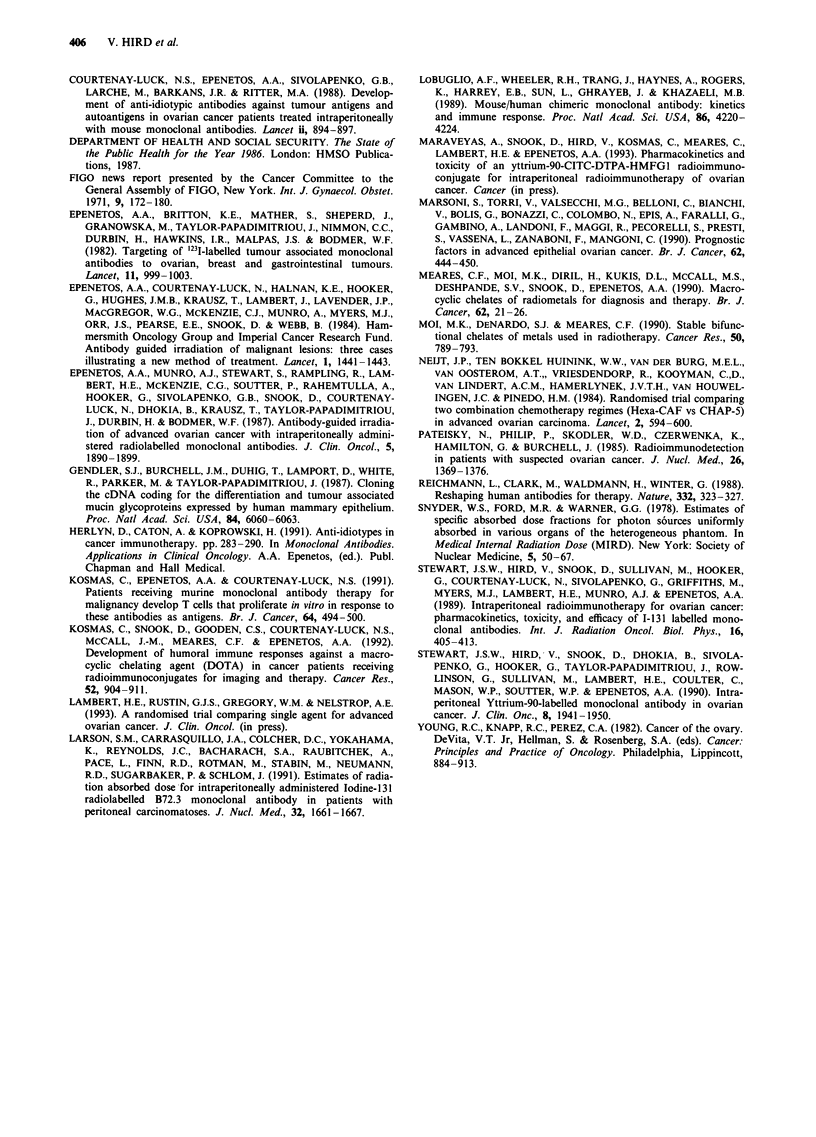# Adjuvant therapy of ovarian cancer with radioactive monoclonal antibody.

**DOI:** 10.1038/bjc.1993.349

**Published:** 1993-08

**Authors:** V. Hird, A. Maraveyas, D. Snook, B. Dhokia, W. P. Soutter, C. Meares, J. S. Stewart, P. Mason, H. E. Lambert, A. A. Epenetos

**Affiliations:** Department of Clinical Oncology, Royal Postgraduate Medical School, Hammersmith Hospital, London, UK.

## Abstract

Fifty-two patients with epithelial ovarian cancer were treated with yttrium-90-labelled monoclonal antibody HMFG1 administered intraperitoneally following conventional surgery and chemotherapy as part of an extended phase I-II trial. The treatment was well tolerated and the only significant toxicity observed was reversible myelosuppression as previously described. Following conventional surgery and chemotherapy, 21 out of the 52 patients had no evidence of residual disease and were regarded as receiving treatment in an adjuvant setting. To date, two of these patients have died of their disease (follow-up 3-62 months, median follow-up 35 months). This extended phase I-II study suggests that patients with advanced ovarian cancer who achieve a complete remission following conventional therapy may benefit from further treatment with intraperitoneal radioactive monoclonal antibody.


Br. J. Cancer (1993), 68, 403–406 © Macmillan Press Ltd., 1993

### Adjuvant therapy of ovarian cancer with radioactive monoclonal antibody

V. Hird[1,2,3], A. Maraveyas[1,3], D. Snook[1], B. Dhokia[1], W.P. Soutter[4], C. Meares[5], J.S.W. Stewart[3], P. Mason[2], H.E. Lambert[3] & A.A. Epenetos[1,3]

[1]*Imperial Cancer Research Fund Oncology Group, Department of Clinical Oncology, Royal Postgraduate Medical School, Hammersmith Hospital, London;* [2]*Department of Obstetrics and Gynaecology, St Mary's Hospital, London;* [3]*Department of Clinical Oncology, Royal Postgraduate Medical School, Hammersmith Hospital, London;* [4]*Department of Obstetrics and Gynaecology, Royal Postgraduate Medical School, Hammersmith Hospital, London;* [4]*Department of Obstetrics and Gynaecology, Hammersmith Hospital, London, UK;* [5]*Department of Chemistry, University of California at Davies, USA.*

**Summary** Fifty-two patients with epithelial ovarian cancer were treated with yttrium-90-labelled monoclonal antibody HMFG1 administered intraperitoneally following conventional surgery and chemotherapy as part of an extended phase I–II trial.

The treatment was well tolerated and the only significant toxicity observed was reversible myelosuppression as previously described. Following conventional surgery and chemotherapy, 21 out of the 52 patients had no evidence of residual disease and were regarded as receiving treatment in an adjuvant setting. To date, two of these patients have died of their disease (follow-up 3–62 months, median follow-up 35 months).

This extended phase I–II study suggests that patients with advanced ovarian cancer who achieve a complete remission following conventional therapy may benefit from further treatment with intraperitoneal radioactive monoclonal antibody.

Cancer of the ovary ranks sixth as a fatal form of cancer in women (Young *et al.*, 1982). Its incidence is approximately 20 per 100,000 with 4,500 new cases and 3,700 deaths per annum in the United Kingdom (Department of Health 1987). At diagnosis most patients have tumour outside the pelvis and this probably accounts for the poor prognosis (FIGO news report 1971). Advances in cytoreductive surgery and postoperative chemotherapy in the last decade have produced response rates of 65–80% but only a small improvement in overall survival (Neijt *et al.*, 1984). Unfortunately, most patients relapse and die of their disease indicating that benefits from surgery and chemotherapy, whether these may be new drugs or new combination of old drugs have reached a plateau (Marsoni *et al.*, 1990).

More than 90% of epithelial ovarian tumours express high levels of many antigens (Bast *et al.*, 1991), including one in particular, known as polymorphic epithelial mucin (PEM) (Gendler *et al.*, 1987). PEM can be described as a 'tumour associated antigen' because although expressed extensively by many epithelial cancers it can also be found at low levels on many normal tissues (Arklie *et al.*, 1981). Several monoclonal antibodies to this antigen and its various epitopes have been made and used for *in vitro* and *in vivo* diagnosis of many cancers including ovarian cancer (Epenetos *et al.*, 1982; Pateisky *et al.*, 1985; Colcher *et al.*, 1983). Since 1983, we have been investigating the possibility of tumour targeting and therapy by the intraperitoneal administration of radiolabelled monoclonal antibodies in patients with ovarian cancer (Epenetos *et al.*, 1984).

We have previously described extensively the pharmacokinetics, biodistribution and toxicity of iodine-131 and yttrium-90-labelled monoclonal antibodies for the treatment of ovarian cancer (Epenetos *et al.*, 1987; Stewart *et al.*, 1989, 1990; Maraveyas *et al.*, 1993). In this report we present the first comprehensive survival data of patients treated in this way from October 1987 to December 1992. Based on our results we propose that this novel modality should now be considered further as a form of adjuvant in patients with cancer of the ovary.

Correspondence: A.A. Epenetos, ICRF Oncology Group, Department of Clincal Oncology, Royal Postgraduate Medical School, Hammersmith Hospital, London W12 0HS, UK.
Received 2 November 1992; and in revised form 1 April 1993.

### Patients, materials and methods

### Patients

Fifty-two patients with known epithelial cancer received intraperitoneal radioimmunotherapy with yttrium-90-labelled monoclonal antibody HMFG1. Patients' ages ranged from 29–76 years. All had performance status above WHO Grade 2. All patients had previously undergone cytoreductive surgery, and all but one were subsequently treated with cisplatin or carboplatin based chemotherapy. One patient (Stage Ic) did not receive chemotherapy. Table I shows the stage and disease status at presentation of all treated patients and Table Ib shows the histology and stage of patients treated as adjuvant, as assessed at second look laparoscopy. It can be seen that there are 22 patients who had no evidence of disease at the time of laparoscopy. One (Stage 1a) was disease free following chemotherapy for relapse and the remaining 21 were regarded as receiving treatment in an adjuvant setting.

### Monoclonal antibody

The monoclonal antibody used in this study was Human Milk Fat Globule 1 (HMFG1) (ICRF, London and Unipath (UK) Ltd, Bedford). HMFG1 is a mouse IgG1 monoclonal antibody that binds to the PEM molecule found on more than 90% of epithelial ovarian carcinomas (Arklie *et al.*, 1981). Patients received 25 mg of antibody.

### Antibody labelling

Yttrium-90 (AERE Harwell, UK) was chelated to the antibody-DTPA, CITC-DTPA or -DOTA conjugate as previously described (Stewart *et al.*, 1990; Meares *et al.*, 1990). Free radioisotope was removed by sephadex G50 gel filtration using phosphate buffered saline as elution buffer. Specific activity of radiolabelled antibody was $\leqslant 5\,Ci\,mg^{-1}$. The final dose of administered antibody was made up to 25 mg of total IgG by adding unlabelled HMFG1 IgG to the radiolabelled fraction. Antibody immunoreactivity was tested in an enzyme-linked immunosorbant assay (ELISA method) before and after radiolabelling and was compared with underivatised antibody using micro titre plates coated with purified antigen. No obvious reduction in immunoreactivity was seen. The administered dose of radioactivity was measured in a SIEL isotope calibration chamber that had been calibrated with an yttrium-90 source (Stewart *et al.*, 1990).

**Table Ia** Patient's number, FIGO stage and extent of disease at antibody treatment

| *Patient no.* | *FIGO stage at presentation* | *Disease stage at antibody therapy* |
|---|---|---|
| 1 | 1a | No evidence of disease following relapse |
| 2 | 1a | Bulky[a] disease + ascites |
| 3 | 1c | No evidence of disease |
| 4 | 1c | No evidence of disease |
| 5 | 1c | Positive peritoneal washings |
| 6 | 1c | No evidence of disease |
| 7 | 2a | Unassessable adhesions |
| 8 | 2b | No evidence of disease |
| 9 | 2b | No evidence of disease |
| 10 | 2c | No evidence of disease |
| 11 | 2c | No evidence of disease |
| 12 | III | No evidence of disease |
| 13 | III | No evidence of disease |
| 14 | III | Unassessable adhesions |
| 15 | III | Bulky disease |
| 16 | III | Bulky disease |
| 17 | III | Bulky disease |
| 18 | III | Bulky disease |
| 19 | III | Minimal[b] disease |
| 20 | III | Minimal disease |
| 21 | III | Bulky disease |
| 22 | III | Bulky disease |
| 23 | III | Bulky disease |
| 24 | III | No evidence of disease |
| 25 | III | Bulky disease |
| 26 | III | Minimal disease |
| 27 | III | Unassessable adhesions |
| 28 | III | Bulky disease |
| 29 | III | No evidence of disease |
| 30 | IV | No evidence of disease |
| 31 | IV | No evidence of disease |
| 32 | IV | Extra peritoneal disease |
| 33 | Ic | Bulky disease |
| 34 | III | No evidence of disease |
| 35 | III | Bulky disease |
| 36 | III | Minimal disease |
| 37 | III | Bulky disease |
| 38 | III | Minimal disease |
| 39 | IV | Bulky disease |
| 40 | III | Bulky disease |
| 41 | III | Minimal disease |
| 42 | IIa | No evidence of disease |
| 43 | Ic | No evidence of disease |
| 44 | IIc | No evidence of disease |
| 45 | III | No evidence of disease |
| 46 | III | Unassessable |
| 47 | Ic | No evidence of disease |
| 48 | III | Unassessable |
| 49 | IV | Bulky disease |
| 50 | III | No evidence of disease |
| 51 | III | No evidence of disease |
| 52 | III | No evidence of disease |

[a]Bulky disease = $>2$ cm. [b]Minimal disease = $<2$ cm.

**Table Ib** FIGO stage and histology of patients treated in an adjuvant setting

| *No.* | *Stage at presentation* | *Histology* |
|---|---|---|
| 1 | Ic | Endometrioid |
| 2 | Ic | Endometrioid |
| 3 | Ic | Serous |
| 4 | IIb | Undifferentiated |
| 5 | IIb | Serous |
| 6 | IIc | Undifferentiated |
| 7 | IIc | Endometrioid |
| 8 | III | Endometrioid |
| 9 | III | Endometrioid |
| 10 | III | Undifferentiated |
| 11 | III | Endometrioid |
| 12 | IV | Undifferentiated |
| 13 | IV | Serous |
| 14 | III | Serous cystadenocarcinoma |
| 15 | IIa | Clear cell |
| 16 | Ic | Serous cystadenocarcinoma |
| 17 | IIc | Well differentiated |
| 18 | III | Serous |
| 19 | Ic | Undifferentiated |
| 20 | III | Serous |
| 21 | III | Endometrioid |

### *Treatment*

A peritoneal dialysis catheter was inserted into the peritoneal cavity during laparoscopy. Open laparoscopy also allowed visual assessment of disease volume, including peritoneal lavage with normal saline for cytological assessment. Minor adhesions, particularly around the liver, were noted in some patients. A good view was obtained in all but five patients, however, all patients were treated. Following the laparoscopy, yttrium-90-labelled antibody (dose range 5.00–30.000 mCi) was infused into the peritoneal cavity with 1.5 litres normal saline or Hartman's solution and the peritoneal dialysis catheter was removed. The patient position was altered every 20 min for the first 2 h to encourage an even distribution of antibody. Patients were nursed in a radiation controlled area for 5 days, during which time blood and urine samples were counted in order to monitor the blood levels of radioactivity and urinary excretion of the radioisotope, respectively.

### *Pharmacokinetics*

Pharmacokinetics, toxicity and dosimetry have been previously reported (Epenetos *et al.*, 1987; Stewart *et al.*, 1989, 1990; Maraveyas *et al.*, 1993). Approximately 30% of the intraperitoneally injected immunoconjugate was absorbed into the systemic circulation by 48 h after administration (Stewart *et al.*, 1989, 1990; Maraveyas *et al.*, 1993).

### Results

### *Toxicity*

The treatment was well tolerated by all patients. Reversible myelosuppression was observed at high doses ($>15$ mCi of HMFG1-DTPA-$^{90}$Y). This toxicity was reduced considerably by the subsequent use of more stable chelating agents known as DOTA and CITC-DTPA (Moi *et al.*, 1990). No significant myelotoxicity was observed even at higher doses of up to 20 mCi of HMFG1-DOTA-$^{90}$Y (Kosmas *et al.*, 1992) and 34 mCi of HMFG1-CITC-DTPA-$^{90}$Y. A correlation between body surface and CITC-DTPA-$^{90}$Y dose was found (Maraveyas *et al.*, 1993). DOTA is potentially immunogenic in patients (Kosmas *et al.*, 1992) as three out of six patients treated with HMG1-DOTA-$^{90}$Y conjugate developed serum sickness reactions manifested as superficial and self limiting skin rashes 10–12 days after treatment. It was also found that treated patients developed anti-DOTA (Kosmas *et al.*, 1990) and anti-CITC-DTPA antibodies. All patients developed human antimouse antibodies as previously reported (Epenetos *et al.*, 1987; Stewart *et al.*, 1989). The difference in toxicity and immunogenicity between DTPA and DOTA linkage between antibody and radionuclide as well as the HAMA levels are reported elsewhere (Kosmas *et al.*, 1992; Maraveyas *et al.*, 1993).

### *Survival*

Figure 1 shows the survival data of the subgroup of 15 patients treated regarded as receiving adjuvant treatment and compares it with a similar group (70 patients) from the same centre (North Thames Ovarian Group). This group comprises of patients who presented with Stage 11b disease or worse and had no evidence of residual disease at laparoscopy following conventional treatment with surgery and chemotherapy. These data show a remarkable difference in survival between the group treated as adjuvant with antibody and the historical control from the North Thames Ovarian Group (Lambert *et al.*, 1993). However, this is not the result

**Figure 1** Actuarial survival of patients treated in an adjuvant setting with monoclonal antibodies. In this Figure a comparison is made of the 15 patients (Stage IIb or greater) adjuvant group treated with antibody and a group of 70 patients from the North Thames ovarian Group who presented with at least Stage IIb disease and who had no evidence of disease at second look laparoscopy. These patients were further randomised to receive further chemotherapy or whole abdominal radiotherapy (Lambert *et al.*, 1993). A further six patients who had stage Ic-IIa disease and were treated similarly with radiolabelled monoclonal antibodies are not shown on this graph. One patient of this group has died.

of a randomised trial, and the patient numbers are small. Survival after antibody therapy of patients with bulky disease treated with radiolabelled antibody is: median survival of 11 months (range 2–31 months), with four patients still alive.

### Discussion

The application of radiolabelled antibodies as specific cytotoxic drugs against cancer has many attractions including selectivity against tumour cells, irradiation of adjacent tumour cells, lack of major side effects and simplicity of radiolabelling and administration. Although tested extensively over the last decade, radiolabelled and other immunoconjugates have had only limited success as anticancer agents.

For the first time, this study demonstrates that radiolabelled antibodies used in an adjuvant setting may reduce the rate of recurrence from ovarian cancer and improve the long term survival. Although survival data from this study appear superior to previously reported studies (Neijt *et al.*, 1984; Marsoni *et al.*, 1990), the patient numbers are small and need to be substantiated by larger phase III randomised studies. Furthermore, because this was a phase I–II study, our cases included a mixture of stages from Ic-IV.

The mechanisms for the action of antibody therapy are not clear from this trial. The calculated doses of radiation delivered by the radioactive antibody are thought to be insufficient for a cytotoxic effect based on calculations using conventional dosimetry tables (Snyder *et al.*, 1978) although more recent studies suggest that higher doses can be delivered (Larson *et al.*, 1991). Unfortunately, there are no comprehensive data on the therapeutic efficacy of radioactive yttrium colloid alone given intraperitoneally after chemotherapy. An alternative possibility is that HMFG1 murine monoclonal antibody when administered intraperitoneally into humans, can cause a cascade of immunological reactions leading to humoral (Courtenay-Luck *et al.*, 1988; Herlyn *et al.*, 1991) and cellular (Kosmas *et al.*, 1991) activation of the immune system with resultant antitumour effects. If this is the cause of the observed prolongation of survival in patients with ovarian cancer in this study, then, ironically, the use of murine monoclonal antibodies may be more effective than the recently described chimeric (LoBuglio *et al.*, 1989), humanised (Reichmann *et al.*, 1988) or completely human (Borrebaeck *et al.*, 1988) monoclonal antibodies.

In summary, this study provides encouragement to the concept of adjuvant therapy with monoclonal antibodies in patients with epithelial ovarian cancer who have no evidence of residual disease after initial surgery and chemotherapy.

We are grateful to the following: D. Allen, R. Biruls, C. Coulter, R. Chandler and J. Taylor-Papadimitriou.

### References

ARKLIE, J., TAYLOR-PAPADIMITRIOU, J., BODMER, W.F., EGAN, M. & MILLIS, R. (1981). Differentiation antigens expressed by epithelial cells in the lactating breast are also detectable in breast cancers. *Int. J. Cancer*, **28**, 23–27.

BAST, R.C. Jr, KNAUF, S., EPENETOS, A., DHOKIA, B., DALY, L., TANNER, M., SOPER, J., CREAMAN, N., GALL, S., KNAPP, R.C., ZURAWSKI, V.R. Jr, SCHLOM, J., KUFE, D.W. & RITTS, R.E. Jr (1991). Coordinate elevation of serum markers in ovarian cancer but not in benign disease. *Cancer*, **68**, 1758–1763.

BORREBAECK, C.A.K., DANIELSSON, K. & MOLLER, S. (1988). Human monoclonal antibodies produced by primary *in vitro* immunization of peripheral blood lymphocytes. *Proc. Natl Acad. Sci. USA*, **85**, 3995–3999.

COLCHER, D., ZALUTSKY, M., KAPLAN, W., KUFE, D., AUSTIN, F. & SCHLOM, J. (1983). Radiolocalization of human mammary tumours in athymic mice by a monoclonal antibody. *Cancer Res.*, **43**, 736–742.

COURTENAY-LUCK, N.S., EPENETOS, A.A., SIVOLAPENKO, G.B., LARCHE, M., BARKANS, J.R. & RITTER, M.A. (1988). Development of anti-idiotypic antibodies against tumour antigens and autoantigens in ovarian cancer patients treated intraperitoneally with mouse monoclonal antibodies. *Lancet* **ii**, 894–897.

DEPARTMENT OF HEALTH AND SOCIAL SECURITY. *The State of the Public Health for the Year 1986*. London: HMSO Publications, 1987.

FIGO news report presented by the Cancer Committee to the General Assembly of FIGO, New York. *Int. J. Gynaecol. Obstet.* 1971, **9**, 172–180.

EPENETOS, A.A., BRITTON, K.E., MATHER, S., SHEPERD, J., GRANOWSKA, M., TAYLOR-PAPADIMITRIOU, J., NIMMON, C.C., DURBIN, H., HAWKINS, I.R., MALPAS, J.S. & BODMER, W.F. (1982). Targeting of $^{123}$I-labelled tumour associated monoclonal antibodies to ovarian, breast and gastrointestinal tumours. *Lancet*, **11**, 999–1003.

EPENETOS, A.A., COURTENAY-LUCK, N., HALNAN, K.E., HOOKER, G., HUGHES, J.M.B., KRAUSZ, T., LAMBERT, J., LAVENDER, J.P., MACGREGOR, W.G., MCKENZIE, C.J., MUNRO, A., MYERS, M.J., ORR, J.S., PEARSE, E.E., SNOOK, D. & WEBB, B. (1984). Hammersmith Oncology Group and Imperial Cancer Research Fund. Antibody guided irradiation of malignant lesions: three cases illustrating a new method of treatment. *Lancet*, **1**, 1441–1443.

EPENETOS, A.A., MUNRO, A.J., STEWART, S., RAMPLING, R., LAMBERT, H.E., MCKENZIE, C.G., SOUTTER, P., RAHEMTULLA, A., HOOKER, G., SIVOLAPENKO, G.B., SNOOK, D., COURTENAY-LUCK, N., DHOKIA, B., KRAUSZ, T., TAYLOR-PAPADIMITRIOU, J., DURBIN, H. & BODMER, W.F. (1987). Antibody-guided irradiation of advanced ovarian cancer with intraperitoneally administered radiolabelled monoclonal antibodies. *J. Clin. Oncol.*, **5**, 1890–1899.

GENDLER, S.J., BURCHELL, J.M., DUHIG, T., LAMPORT, D., WHITE, R., PARKER, M. & TAYLOR-PAPADIMITRIOU, J. (1987). Cloning the cDNA coding for the differentiation and tumour associated mucin glycoproteins expressed by human mammary epithelium. *Proc. Natl Acad. Sci. USA*, **84**, 6060–6063.

HERLYN, D., CATON, A. & KOPROWSKI, H. (1991). Anti-idiotypes in cancer immunotherapy. pp. 283–290. In *Monoclonal Antibodies. Applications in Clinical Oncology*. A.A. Epenetos, (ed.). Publ. Chapman and Hall Medical.

KOSMAS, C., EPENETOS, A.A. & COURTENAY-LUCK, N.S. (1991). Patients receiving murine monoclonal antibody therapy for malignancy develop T cells that proliferate *in vitro* in response to these antibodies as antigens. *Br. J. Cancer*, **64**, 494–500.

KOSMAS, C., SNOOK, D., GOODEN, C.S., COURTENAY-LUCK, N.S., MCCALL, J.-M., MEARES, C.F. & EPENETOS, A.A. (1992). Development of humoral immune responses against a macrocyclic chelating agent (DOTA) in cancer patients receiving radioimmunoconjugates for imaging and therapy. *Cancer Res.*, **52**, 904–911.

LAMBERT, H.E., RUSTIN, G.J.S., GREGORY, W.M. & NELSTROP, A.E. (1993). A randomised trial comparing single agent for advanced ovarian cancer. *J. Clin. Oncol.* (in press).

LARSON, S.M., CARRASQUILLO, J.A., COLCHER, D.C., YOKAHAMA, K., REYNOLDS, J.C., BACHARACH, S.A., RAUBITCHEK, A., PACE, L., FINN, R.D., ROTMAN, M., STABIN, M., NEUMANN, R.D., SUGARBAKER, P. & SCHLOM, J. (1991). Estimates of radiation absorbed dose for intraperitoneally administered Iodine-131 radiolabelled B72.3 monoclonal antibody in patients with peritoneal carcinomatoses. *J. Nucl. Med.*, **32**, 1661–1667.

LOBUGLIO, A.F., WHEELER, R.H., TRANG, J., HAYNES, A., ROGERS, K., HARREY, E.B., SUN, L., GHRAYEB, J. & KHAZAELI, M.B. (1989). Mouse/human chimeric monoclonal antibody: kinetics and immune response. *Proc. Natl Acad. Sci. USA*, **86**, 4220–4224.

MARAVEYAS, A., SNOOK, D., HIRD, V., KOSMAS, C., MEARES, C., LAMBERT, H.E. & EPENETOS, A.A. (1993). Pharmacokinetics and toxicity of an yttrium-90-CITC-DTPA-HMFG1 radioimmunoconjugate for intraperitoneal radioimmunotherapy of ovarian cancer. *Cancer* (in press).

MARSONI, S., TORRI, V., VALSECCHI, M.G., BELLONI, C., BIANCHI, V., BOLIS, G., BONAZZI, C., COLOMBO, N., EPIS, A., FARALLI, G., GAMBINO, A., LANDONI, F., MAGGI, R., PECORELLI, S., PRESTI, S., VASSENA, L., ZANABONI, F., MANGONI, C. (1990). Prognostic factors in advanced epithelial ovarian cancer. *Br. J. Cancer*, **62**, 444–450.

MEARES, C.F., MOI, M.K., DIRIL, H., KUKIS, D.L., MCCALL, M.S., DESHPANDE, S.V., SNOOK, D., EPENETOS, A.A. (1990). Macrocyclic chelates of radiometals for diagnosis and therapy. *Br. J. Cancer*, **62**, 21–26.

MOI, M.K., DENARDO, S.J. & MEARES, C.F. (1990). Stable bifunctional chelates of metals used in radiotherapy. *Cancer Res.*, **50**, 789–793.

NEIJT, J.P., TEN BOKKEL HUININK, W.W., VAN DER BURG, M.E.L., VAN OOSTEROM, A.T.,, VRIESDENDORP, R., KOOYMAN, C.,D., VAN LINDERT, A.C.M., HAMERLYNEK, J.V.T.H., VAN HOUWELINGEN, J.C. & PINEDO, H.M. (1984). Randomised trial comparing two combination chemotherapy regimes (Hexa-CAF vs CHAP-5) in advanced ovarian carcinoma. *Lancet*, **2**, 594–600.

PATEISKY, N., PHILIP, P., SKODLER, W.D., CZERWENKA, K., HAMILTON, G. & BURCHELL, J. (1985). Radioimmunodetection in patients with suspected ovarian cancer. *J. Nucl. Med.*, **26**, 1369–1376.

REICHMANN, L., CLARK, M., WALDMANN, H., WINTER, G. (1988). Reshaping human antibodies for therapy. *Nature*, **332**, 323–327.

SNYDER, W.S., FORD, M.R. & WARNER, G.G. (1978). Estimates of specific absorbed dose fractions for photon sóurces uniformly absorbed in various organs of the heterogeneous phantom. In *Medical Internal Radiation Dose* (MIRD). New York: Society of Nuclear Medicine, **5**, 50–67.

STEWART, J.S.W., HIRD, V., SNOOK, D., SULLIVAN, M., HOOKER, G., COURTENAY-LUCK, N., SIVOLAPENKO, G., GRIFFITHS, M., MYERS, M.J., LAMBERT, H.E., MUNRO, A.J. & EPENETOS, A.A. (1989). Intraperitoneal radioimmunotherapy for ovarian cancer: pharmacokinetics, toxicity, and efficacy of I-131 labelled monoclonal antibodies. *Int. J. Radiation Oncol. Biol. Phys.*, **16**, 405–413.

STEWART, J.S.W., HIRD, V., SNOOK, D., DHOKIA, B., SIVOLAPENKO, G., HOOKER, G., TAYLOR-PAPADIMITRIOU, J., ROWLINSON, G., SULLIVAN, M., LAMBERT, H.E., COULTER, C., MASON, W.P., SOUTTER, W.P. & EPENETOS, A.A. (1990). Intraperitoneal Yttrium-90-labelled monoclonal antibody in ovarian cancer. *J. Clin. Onc.*, **8**, 1941–1950.

YOUNG, R.C., KNAPP, R.C., PEREZ, C.A. (1982). Cancer of the ovary. DeVita, V.T. Jr, Hellman, S. & Rosenberg, S.A. (eds). *Cancer: Principles and Practice of Oncology*. Philadelphia, Lippincott, 884–913.